# Mitogenomic features of the citrus leafminer, *Phyllocnistis citrella* (Lepidoptera: Gracillariidae) and the related mitogenomic phylogeny

**DOI:** 10.1080/23802359.2020.1787897

**Published:** 2020-07-14

**Authors:** Hong-Ling Liu, Zhi-Teng Chen, Song Chen, Qing-Dong Chen, De-Qiang Pu, Yue-Yue Liu, Xu Liu

**Affiliations:** a Institute of Plant Protection, Sichuan Academy of Agricultural Sciences, Key Laboratory of Integrated Pest Management of Southwest Crops, Ministry of Agriculture, Chengdu, China; b School of Grain Science and Technology, Jiangsu University of Science and Technology, Zhenjiang, China; c Analysis and Testing Center of Sichuan Academy of Agricultural Sciences, Chengdu, China

**Keywords:** Lepidoptera, Gracillariidae, mitogenome, phylogeny

## Abstract

The complete mitochondrial genome of the citrus leafminer, *Phyllocnistis citrella* (Lepidoptera: Gracillariidae) was sequenced for the first time, which was 15,416 bp in length and with an A + T content of 81.9%. The common set of 37 genes (13 PCGs, 22 tRNA genes, and two rRNA genes) were all annotated. Most PCGs had standard ATN start codons and TAN stop codons. Most tRNA genes exhibited typical cloverleaf secondary structures, whereas the dihydrouridine (DHU) arm of *trnSer (AGN)* was reduced. The mitochondrial phylogenetic analyses consistently recovered the relationships within Gracillariidae.

The citrus leafminer (CLM), *Phyllocnistis citrella* Stainton 1856 (Lepidoptera: Gracillariidae), is a widespread pest species of citrus and related Rutaceae in Asia. Accumulation of new molecular data of *P. citrella* is a vital step to find new control strategies for this pest from a genetic view. However, the mitogenome of *P. citrella* is still unknown, which is a barrier for us to understand the relationship between *P. citrella* and its related lepidopteran pests. In this study, the complete mitogenome *P. citrella* is determined and reported for the first time. The sequence is accessible in GenBank with the accession number MN792920. The mitogenomic structure, nucleotide compositions, codon usages of PCGs, secondary structures of tRNA genes and the control region were analyzed to better understand the genetic characters of this important pest. The mitogenomic phylogeny of Gracillariidae was also investigated based on the newly obtained data.

The male adult samples of *P. citrella* were collected from Nanchong City, Sichuan Province of China (30.82 N, 106.09 E) in June 2019; all specimens and DNA samples are stored in the Insect Collection of Sichuan Academy of Agricultural Sciences (ICSAAS No. ICSAAS-LEP-1), Chengdu, China. The complete mitogenome of *P. citrella* is a typical double-strand circular molecule with 15,416 base pairs and the standard set of 37 genes (13 PCGs, 22 tRNA genes, and two rRNA genes). The only control region of *P. citrella* is short with only 405 bp, but its A + T content is the highest (94.1%) in the mitogenome. The mitochondrial genes of *P. citrella* are rearranged, exhibiting the *trnMet*-*trnIle*-*trnGln* rearrangement. The whole mitogenome of *P. citrella* is strongly biased toward A and T nucleotides (81.9%) with negative AT-skew and GC-skew values. Most PCGs utilize the standard ATN start codon except for the special start codon CGA used by *cox1*. Most PCGs terminate with the complete stop codon TAA, but *cox1*, *cox2*, and *cox3* end with an incomplete stop codon T. Most of the tRNA genes can fold into cloverleaf secondary structures, but the TΨC loops of *trnPhe* and *trnTyr* are reduced and the dihydrouridine (DHU) arm of *trnSer* is reduced into a small loop. The large ribosomal RNA (*rrnL*) gene is 1370 bp in length, with an A + T content of 85.8%. The small ribosomal RNA (*rrnS*) gene is 761-bp long, with an A + T content of 86.1%.

The phylogenetic position of *P. citrella* in Gracillariidae was investigated using BI and ML analyses, which generated identical topological structures (Figure 1). In both trees, *P. citrella* was closely grouped with other members of Gracillariidae. The two species of *Phyllonorycter* were grouped in the same clade, indicating the efficiency of mitogenome data in phylogenetic reconstructions. The two families, Psychidae and Tineidae were supported as sister groups in Tineoidea. Each family studied herein was recovered as monophyletic with high support (posterior probability = 1, bootstrap value = 100). The reconstructed topology of the three families is identical with the results of Robinson (1988) based on morphological characters, and consistent with the mitogenomic phylogeny of Jeong et al. (2018) and Chen et al. (2019).

**Figure 1.**
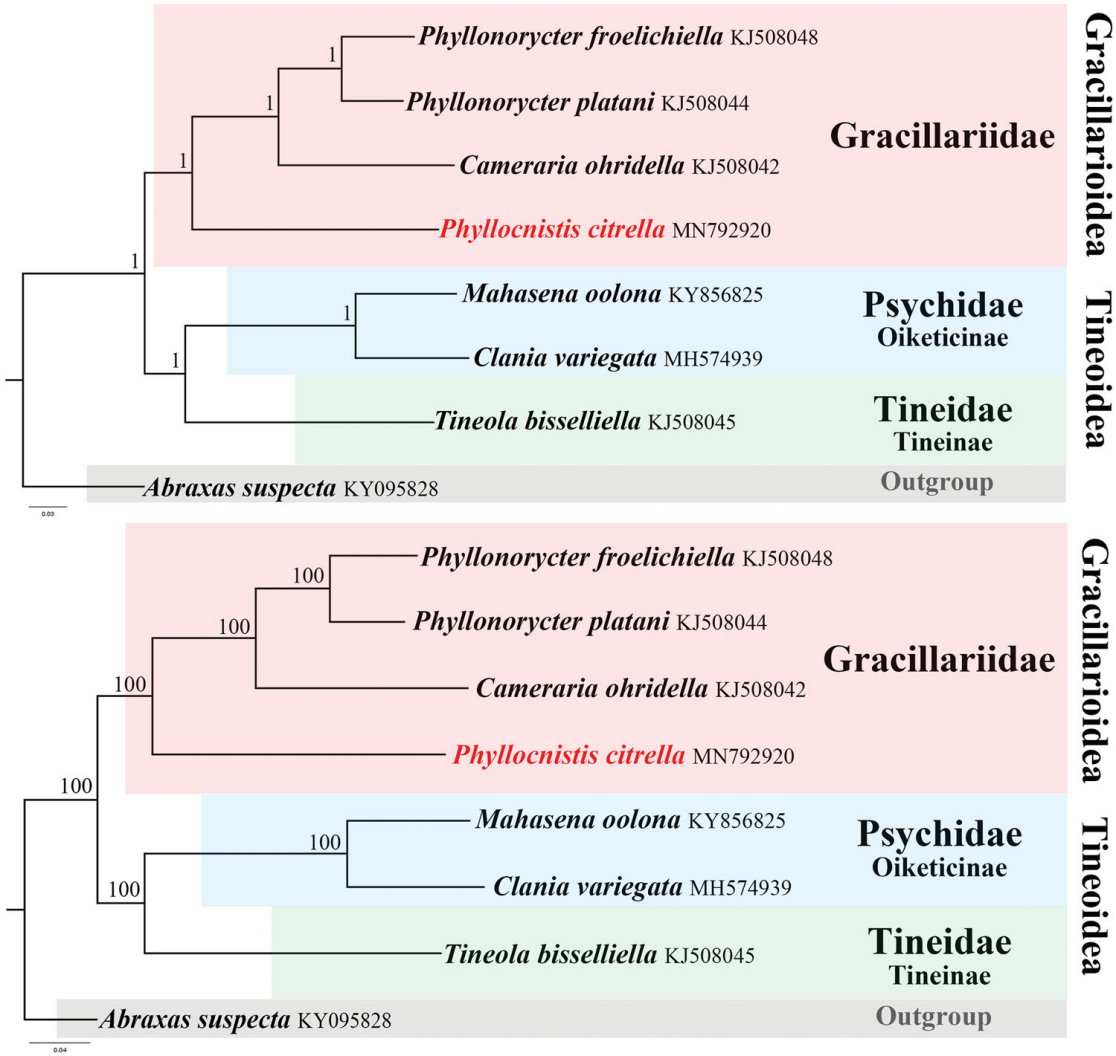
Phylogenetic relationships of eight sequenced lepidopteran species. Numbers at the nodes of the upper BI tree are posterior probabilities; numbers at the nodes of the second ML tree are bootstrap values. The GenBank accession numbers are indicated after the scientific names.

## Data Availability

The data that support the findings of this study are openly available in ‘NCBI’ at https://www.ncbi.nlm.nih.gov/, reference number MN792920.
